# The safety and efficacy of high versus low vancomycin trough levels in the treatment of patients with infections caused by methicillin-resistant *Staphylococcus aureus*: a meta-analysis

**DOI:** 10.1186/s13104-016-2252-7

**Published:** 2016-09-29

**Authors:** Sasima Tongsai, Pornpan Koomanachai

**Affiliations:** 1 Clinical Epidemiology Unit, Office for Research and Development, Faculty of Medicine, Siriraj Hospital, Mahidol University, 2 Wang Lang Road, Siriraj, Bangkoknoi, Bangkok, 10700 Thailand; 2 Division of Infectious Diseases and Tropical Medicine, Faculty of Medicine, Siriraj Hospital, Mahidol University, 2 Wang Lang Road, Siriraj, Bangkoknoi, Bangkok, 10700 Thailand

**Keywords:** Vancomycin trough levels, Clinical success, Mortality, Nephrotoxicity, Methicillin-resistant *Staphylococcus aureus*, Meta-analysis

## Abstract

**Background:**

Recent guidelines have recommended vancomycin trough levels of 15–20 mg/L for treatment of serious infections caused by methicillin-resistant *Staphylococcus aureus* (MRSA). However, high trough levels may increase risk of nephrotoxicity and mortality, and high vancomycin trough levels have not been well studied. This study was designed to combine safety and efficacy results from independent studies and to compare between high and low vancomycin trough levels in the treatment of MRSA-infected patients using meta-analysis.

**Methods:**

From 19 eligible studies, 9 studies were included in meta-analysis to compare clinical success between high and low vancomycin trough levels, while 10 and 11 studies met criteria for comparing trough levels and nephrotoxicity and trough levels and mortality, respectively. The PubMed/Medline, Web of Science, and Scopus databases, and hand searching were used to identify eligible studies dated up to March 2016. Of 2344 subjects with MRSA infection, 1036 were assigned to trough levels ≥15 mg/L and 1308 to trough levels <15 mg/L.

**Results:**

High vancomycin trough levels were found to be associated with risk of nephrotoxicity (odds ratio [OR] 2.14, 95 % confidence interval [CI] 1.42–3.23 and adjusted OR 3.33, 95 % CI 1.91–5.79). There was no evidence of difference between high and low vancomycin trough levels for mortality (OR; 1.09; 95 % CI 0.75–1.60) or clinical success (OR 1.07; 95 % CI 0.68–1.68).

**Conclusion:**

In this study, high vancomycin trough levels were identified as an independent factor associated with risk of nephrotoxicity in MRSA-infected patients. Association between vancomycin trough levels and both adverse effects and clinical outcomes requires further study.

**Electronic supplementary material:**

The online version of this article (doi:10.1186/s13104-016-2252-7) contains supplementary material, which is available to authorized users.

## Background

Vancomycin was first approved for use in 1958 by the US Food and Drug Administration (FDA) for treating penicillin-resistant *Staphylococcus aureus* infection. Vancomycin continues to be widely used, particularly due to recent increases in incidence of serious methicillin-resistant *S. aureus* (MRSA) infections. Although vancomycin has been used for over 40 years, it still remains a standard treatment for infections caused by MRSA. However, reports began to appear in 2003 describing clinical failures of vancomycin treatment due to the emergence of MRSA with reduced vancomycin susceptibility [1, 2]. Since 2003, several similar studies have been published in which vancomycin-susceptible MRSA strains were identified and clinical failure resulted, despite monitoring and maintenance of trough levels in the recommended range to ensure vancomycin efficacy [3, 4]. Since more than two decades ago and according to Clinical and Laboratory Standards Institute (CLSI) guidelines [5, 6], vancomycin MICs have increased over time—a phenomenon that is referred to as vancomycin MIC creep [7, 8]. As a result of published studies demonstrating vancomycin treatment failure in patients with *S*. *aureus* infections who had a vancomycin MIC ≥4 mg/L, the CLSI lowered pre-2006 vancomycin MIC breakpoints by broth microdilution (BMD) from ≤4 to ≤2 µg/mL for susceptible strains of *S. aureus*.

Early target trough levels for vancomycin were 5–10 mg/L, and then they were increased to 8–15 mg/L. Vancomycin trough levels of 15–20 mg/L (area under the curve [AUC]: minimum inhibitory concentration [MIC] ratio ≥400 in most patients if MIC is ≤1 mg/L) are recommended by the Infectious Diseases Society of America (IDSA) and the American Thoracic Society (ATS) in patients with normal renal function and serious infections [9, 10]. New guidelines and expert panel recommendations for vancomycin therapeutic drug monitoring (TDM) recommend trough levels of 15–20 mg/L to prevent development of resistance and improve clinical outcomes. Whether trough levels explain the apparent failure of vancomycin treatment remains controversial. Some studies have shown higher troughs not to be associated with increased vancomycin efficacy in patients with MRSA infections [11–16], while others studies did find association with increased efficacy [17–20]. The guideline suggests that vancomycin efficacy in invasive infections caused by MRSA is determined by adult pharmacokinetic and pharmacodynamic data that achieved AUC/MIC of ≥400 which correlates with vancomycin trough levels of 15–20 μg/mL. Because an AUC/MIC goal value is difficult to calculate and given the good comparability between AUC/MIC and vancomycin trough levels, trough levels are considered to be both the most accurate and the most practical method for therapeutic drug monitoring of vancomycin.

The new guidelines also warn that vancomycin nephrotoxicity should be considered if serum creatinine concentration increases greater than or equal to 0.5 mg/dL, or more than 50 % over the baseline value. Previously, most reports of acute kidney injury (AKI) were likely linked to impurities in vancomycin preparation, which was sometimes disparagingly referred to as ‘Mississippi mud’. However, in the 1960s, the purity of vancomycin preparation increased to 75 % with a further increase in purity to 92–95 % in 1985 [10, 21]. As a result, impurities in vancomycin preparation were no longer a concerned.

A recent meta-analysis found association between high vancomycin trough levels and nephrotoxicity in subjects with Gram-positive infections and in patients with various other types of infections [22]. However, in that meta-analysis, the effect of vancomycin trough levels on nephrotoxicity and clinical outcomes in patients with MRSA infection was not investigated. It also remains unclear whether an increase in vancomycin trough levels could improve clinical outcomes of vancomycin treatment in MRSA infections. As such, the aim of this study was to combine safety and efficacy results from independent studies and to compare between high and low vancomycin trough levels in the treatment of MRSA-infected patients using meta-analysis.

## Methods

### Data sources and search strategy

Multiple electronic databases including MEDLINE/Pubmed, Web of Science, and Scopus, were searched for reports published up to March 2016. The search terms used included “vancomycin”, “trough levels”, “trough concentration”, “nephrotoxicity”, and “methicillin-resistant *S. aureus*”. The word vancomycin was also combined with other terms in various combinations. MeSH terms for “vancomycin” and “methicillin-resistant *Staphylococcus aureus*” were also included in our PubMed search. In addition, a hand search of reference lists of selected studies and grey literature (conference proceedings, dissertations, theses, and reports) was conducted to identify relevant studies not included in electronic databases. Abstract lists and conference proceedings from the 2007 to 2015 scientific meetings of the Infectious Diseases Society of America, International Society for Infectious Diseases, American Society for Microbiology, and European Society of Clinical Microbiology and Infectious Diseases were also searched to identify possibly eligible studies (see Additional file 1). No language restrictions were applied for these searches.

### Study selection

A single investigator (ST) screened the titles and abstracts of potentially eligible studies, and then examined the articles to determine whether they met the established inclusion criteria. Selected articles were then double-checked in detail by the second investigator (PK). In the end, all selected articles were reviewed and approved by both investigators, with no disagreement between investigators regarding the eligibility of an article identified by one or the other investigator. All published and unpublished studies were included if they met the following criteria: (1) evaluated primary outcomes (i.e., nephrotoxicity, mortality and/or clinical success) of adult patients with MRSA infections; and, (2) the observed outcomes could be extracted and classified into two trough levels. Papers were excluded if they were characterized by one or more of the following: (1) conducted in pediatric patients; (2) focused on treatment of MRSA-infected patients with vancomycin MIC of ≥2 mg/L; (3) we could not extract information relevant to only MRSA infections; or, (4) they were reviews, guidelines, editorials, or non-human research.

### Data extraction and management

Data extraction and management for included studies were performed by the first investigator (ST). Both investigators (ST, PK) met to discuss data extraction findings and characteristics of each study for purposes of ensuring clear understanding of assessment criteria. The following information was extracted from each eligible study: the first author’s last name, year of publication, study location, patient characteristic, study design, sample size (number of subjects in high and low trough groups), timing of vancomycin trough level measurement, duration of vancomycin therapy, concomitant nephrotoxic agent, outcomes and definitions of outcomes, ORs and 95 % CIs for each outcome, and covariates adjusted for in multivariable models (when they were available). The first investigator (ST) also screened and double-checked data for data entry errors.

In this meta-analysis, patients were analyzed according to their vancomycin trough levels, which were defined as <15 mg/L for low trough levels and ≥15 mg/L for high trough levels. For each eligible study, categories were recoded as follows: categories “<10” and “10–14 mg/L were collapsed into <15; “15–20 mg/L” was classified as ≥15 mg/L; and, categories “<15” and “≥15 mg/L” were retained in their original category for all analyses. If the information was available, patients with trough level >20 mg/L were eliminated from analysis so we could focus only on the 15–20 mg/L range in high trough patients. For this study, serious MRSA infection was defined as a person with any one or more of the following infections caused by MRSA: bacteremia, endocarditis, osteomyelitis, meningitis, pneumonia, and/or central nervous system infection.

### Data synthesis and analysis

A funnel plot was generated to assess funnel plot asymmetry by plotting the standard error of the odds ratio on the vertical axis and the odds ratio on the horizontal axis, with degree of asymmetry tested by Egger’s test [23] and Begg’s test [24]. A *p* value <0.05 was considered to be statistically significant asymmetry. A forest plot was produced to show the odds ratio with 95 % CI of each study and the pooled odds ratio with the corresponding 95 % CI. Jackknife procedure-based sensitivity analysis was performed by omitting one study at a time to evaluate the effect of individual studies on the stability of the results.

Pooled odds ratio was calculated using the DerSimonian and Laird random-effects model [25]. Greenland-Robin variance formula was used to calculate confidence intervals of the pooled odds ratio. Heterogeneity among studies was evaluated using the Chi square based Q statistics (χ^2^), measure of inconsistency (*I*^2^), and between-study variance (*τ*^*2*^). A *p* value <0.10 was considered to indicate statistically significant heterogeneity while *I*^2^ > 50 % was considered to indicate at least moderate heterogeneity. Trim and fill method [26, 27] was used to estimate overall effect size after adjusting for funnel plot asymmetry arising from publication bias [28]. All analyses were performed using R software with meta package (R Foundation, Vienna, Austria) [29].

## Results

**Fig. 1:**
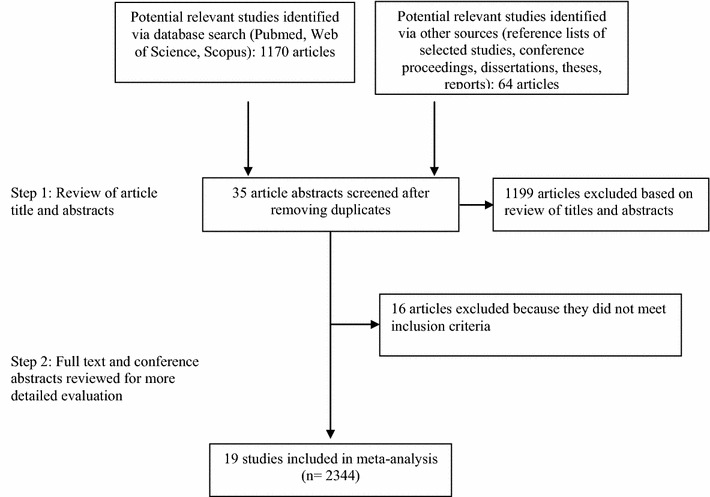
Flow diagram of study selection process

The article selection process for this meta-analysis is shown in Fig. 1. The initial search comprising three online databases, hand searching and grey literature databases for reports published up to March 2016 yielded 1170 articles. That list was narrowed to 35 potentially relevant articles after a review of their titles and abstracts. After a more detailed review, an additional 16 articles were excluded. The remaining 19 reports fully satisfied the inclusion criteria and were included in the final analysis. All included studies were reported in the English language.

Of the 19 included studies, three studies had three categories of trough levels (<15, 15–20 and >20 mg/L), one study had four categories (<10, 10–14.9, 15–20 and >20 mg/L), and the remaining 15 studies had two trough levels (<15 and ≥15 mg/L). One hundred and twelve patients with trough level >20 mg/L were excluded from this meta-analysis. However these patients were later and temporarily included for purposes of comparing outcome results against the results and conclusion with these patients excluded (data not shown). Of 19 eligible studies, meta-analysis of nine studies was per formed to investigate the link between vancomycin trough levels and clinical success, while 10 and 11 studies met the criteria for investigating association between trough levels and nephrotoxicity and trough levels and mortality, respectively. Three studies were available for analysis of the relationship between nephrotoxicity and vancomycin trough levels by combining adjusted OR estimates from multiple logistic regression analysis in order to adjust confounding variables of each included study.

The main characteristics of the studies included in this meta-analysis are presented in Table 1. Included studies were conducted and reported between 1999 and 2013. One thousand and thirty-six subjects were assigned to the high trough group (trough levels ≥15 mg/L) and 1308 subjects were assigned to the low trough group (trough levels <15 mg/L). Effect sizes of outcome measures for each included study are shown in Table 2.

**Table 1:** Summarized characteristics of the eligible studies in the meta-analysis

Author	Country	Study period	Patient characteristic	Study design (No. of subjects)	Timing of vancomycin trough level measurement	Duration of vancomycin therapy
Casapao et al. [65, 66]	Michigan, USA	2004–2012	Patients with MRSA IE infections treated with VAN for ≥3 days, and who had initial VAN trough levels evaluated	Retrospective cohort study (n = 128)	Initial	NA
Zelenitsky et al. [67]	Canada	2002–2007	Patients age ≥18 years treated with VAN for MRSA-associated septic shock	Retrospective study (n = 34)	Initial	
Tadros et al. [68]	Canada	2011	Patients aged ≥18 years with MRSA pneumonia infections treated with VAN, and who had at least one initial VAN trough level measurement	Prospective surveillance study (n = 161)	Average	NA
Arshad et al. [69]	USA	2005–2007	Patients with MRSA bacteraemia treated with VAN, while patients with acute renal failure on admission, dialysis and those not at steady-state after third dose of VAN were excluded	Retrospective study (n = 104)	Initial and average	NA
Leu et al. [34]	Taiwan	2009–2011	Patients with MRSA infections (most infections were pneumonia, wound infection, or bacteremia) treated with VAN for ≥3 days, and who had at least one VAN trough concentration measurement	Non-randomized comparative study (n = 76)	Not stated	NA
Kullar et al. [17]	Michigan, USA	2005–2010	Adult patients with complicated MRSAB infections treated with VAN for ≥72 h	Retrospective quasi-experimental study (n = 200)	Initial	Median durations of VAN were 13 and 8.5 days for patients with trough levels <15 and ≥15 mg/L, respectively
Rojas et al. [38]	Spain	2005–2009	Adult patients with MRSA bloodstream infections treated with VAN, and who had at least one VAN trough concentration measurement	Retrospective cohort study (n = 104)	Average	Median duration of VAN was 14 days (range 8–19) and 42.3% of patients received VAN for >14 days
Wunderink et al. [70]	USA	2004–2010	Patients aged ≥18 years with MRSA nosocomial pneumonia who had an expected survival of ≥72 h	Multicenter prospective study, since there was no randomization with respect to VAN trough levels (n = 333)	Average (Median)	NA
Bosso et al. [71]	South Carolina, USA	2008–2010	Patients aged ≥18 years with MRSA infections treated with VAN for ≥72 h, and who had at least one VAN trough concentration measured 2–4 days into therapy	Multicenter prospective observational study (n = 288)	Initial single trough level measured 2–4 days into therapy; in case of multiple measurements, the average trough level was calculated	Average length of VAN therapy was 9 days (median 7.5)
Chan et al. [72]	USA	2003–2007	Patients aged ≥17 years with MRSA VAP treated with ≥4 days of VAN therapy	Retrospective study (n = 72)	Initial single trough level measured after ≥3 doses of VAN therapy in patients with normal renal function or after a minimum of 5 estimated VAN half lives in patients with renal insufficiency or requiring hemodialysis	Mean durations of VAN were 10.8 days (range 7–23) and 11.6 days (range 7–42 days) for patients with trough levels <15 and ≥15 mg/L, respectively
Choi et al. [13]	Korea	2002–2010	Patients aged ≥15 years with MRSAB who received VAN for >48 h, and who had at least one VAN trough level measurement within 3–7 days into VAN therapy	Retrospective cohort study (n = 73)	Not stated	NA
Chung et al. [36]	Korea	2005–2007	Patients aged ≥18 years in the ICU who had MRSA pneumonia infection treated with VAN for ≥72 h	Prospective study (n = 68)	Initial trough level measured immediately before the next dose after 3–5 doses of VAN therapy	Mean durations of VAN were 15.5 and 12.8 days for patients with trough levels <15 and ≥15 mg/L, respectively
Clemens et al. [37]	USA	2008–2009	Patients aged ≥18 years with MRSAB who received VAN ≥24 h, and who had a documented steady-state VAN trough concentration measurement	Retrospective cohort study (n = 94)	Initial single trough level measured immediately before a scheduled dose after ≥24 h of completing VAN therapy	Average duration of VAN was 14.8 days (range 4–56)
Honda et al. [73]	USA	2005–2007	Patients aged ≥18 years with MRSAB who received VAN as initial therapy, and who had a documented steady-state VAN trough concentration measurement	Prospective cohort study (n=151)	Initial trough level measured before the 4th dose of VAN	NA
Kullar et al. [33]	USA	2005–2010	Adult patients with MRSAB infection who received VAN as initial therapy for ≥72 h	Retrospective cohort study (n = 280)	Initial trough level measured immediately before the 4th dose of VAN	NA
Hermsen et al. [35]	USA	2005–2007	Patients aged ≥19 years with MRSA pneumonia, endocarditis, or osteomyelitis treated with VAN for ≥5 days, and who had at least one VAN trough concentration measurement	Retrospective cohort study (n = 55)	Average	Median durations of VAN were 12 days (P25–P75: 8–18) and 11 days (P25–P75: 7–16) for patients with trough levels <15 and ≥15 mg/L, respectively
Jeffres et al. [31]	USA	1999–2005	All hospitalized patients with MRSA HCAP treated with VAN for ≥72 h	Retrospective cohort study (n = = 94)	Initial trough level measured immediately after the 3rd dose of VAN	NA
Hidayat et al. [30]	California, USA	2004–2005	Patients aged ≥18 years infected with any type of nosocomial MRSA treated with VAN for ≥72 h	Prospective cohort study (n = 95)	Initial and average	Mean durations of VAN were 11.5 days (range 6.5–13.5) and 12 days (range 7–17) for patients with trough levels <15 and ≥15 mg/L, respectively

*MRSA IE* MRSA infective endocarditis, *VAN* vancomycin, *NA* not available, *MRSAB* MRSA bacteremia, *VAP* ventilator associated pneumonia, *ICU* intensive care unit, *P25* 25th percentile, *P75* 75th percentile, *HCAP* health care-associated pneumonia

**Table 2:** Effect size of outcome measures of eligible studies

Authors	Concomitant nephrotoxic agent	Outcome definition	Comparable outcome	High trough group, n (%)	Low trough group, n (%)	*p* value^a^	Crude OR (95% CI)	Adjusted OR (95% CI)
Casapao et al. [65, 66]	NA	Mortality: 30-day all-cause mortality		**n** **=** **61**	**n** **=** **67**			
			Mortality	12 (19.7)	14 (20.9)	0.962	0.93 (0.39–2.20)	NA
		Clinical success: (1) resolution of bacteremia in ≤7 days of VAN therapy; (2) resolution of signs and symptoms; (3) required no change of antibiotics; and, (4) non-recurrence of BSI or survived for at least 30 days		**n** **=** **34**	**n** **=** **75**			
			Clinical success	15 (44.1)	30 (40)	0.846	1.18 (0.52–2.69)	NA
Zelenitsky et al. [67]	NA	Mortality: 30-day all-cause mortality from the date of the first positive blood culture		**n** **=** **17**	**n** **=** **18**			
			Mortality	5 (29.4)	13 (72.2)	0.028	0.16 (0.04–0.69)	NA
Tadros et al. [68]	NA	Mortality: 30-day all-cause mortality		**n** **=** **41**	**n** **=** **56**			
			Mortality	10 (24.4)	11 (19.6)	0.756	1.32 (0.50–3.48)	NA
Arshad et al. [69]	NA	Mortality: Death within 30 days		**n** **=** **49**	**n** **=** **55**			
			Mortality	7 (14.3)	3 (5.5)	0.185	2.89 (0.70–11.86)	NA
		Nephrotoxicity: Increase in S_Cr_ of 0.5 mg/dL or ≥50 % increase from initiation of VAN to 3 days for at least 2 consecutive measurements	Nephrotoxicity	13 (26.5)	5 (9.1)	0.037	3.61 (1.18–11.03)	
		Clinical success: (1) survived for at least 30 days; (2) without recurrence of MRSA infection at least 30 days; and/or, (3) negative blood culture at least 6 days	Clinical success	40 (81.6)	48 (87.3)	0.601	0.65 (0.22–1.90)	
Leu et al. [34]	Not stated	Nephrotoxicity: Increase in S_Cr_ of 0.5 mg/d3L or decrease in CrCl >50 % from baseline		**n** **=** **45**	**n** **=** **31**			
			Nephrotoxicity	10 (22.2)	5 (16.1)	0.717	1.49 (0.45–4.87)	NA
		Clinical cure: All signs and symptoms of infection were resolved after discontinuation of VAN	Clinical cure	11 (24.4)	12 (38.7)	0.282	0.51 (0.19–1.38)	NA
Kullar et al. [17]	Aminoglycoside, colistin, acyclovir	Mortality: 30-day all-cause mortality		**n** **=** **100**	**n** **=** **100**			
			Mortality	13 (13.0)	8 (8.0)	0.356	1.72 (0.68–4.35)	NA
		Nephrotoxicity: increase in S_Cr_ concentrations of 0.5 mg/dL or ≥50% increase from baseline for at least 2–3 consecutive measurements	Nephrotoxicity	18 (18.0)	15 (15.0)	0.703	1.24 (0.59–2.63)	NA
		Clinical success: (1) Survived for at least 30 days; (2) resolution of signs and symptoms of MRSAB at the end of VAN therapy; or, (3) eradication of MRSAB after at least 7 days of VAN therapy	Clinical success	60 (60.0)	45 (45.0)	0.047	1.83 (1.05–3.21)	NA
Rojas et al. [38]	Not stated	Mortality: overall mortality		**n** **=** **24**	**n** **=** **61**			
			Mortality	8 (33.3)	22 (36.1)	0.988	0.89 (0.33–2.40)	NA
Wunderink et al. [70]	Not stated	Nephrotoxicity: increase in S_Cr_ of 0.5 mg/dL if normal at baseline or 50% increase in S_Cr_ if abnormal at baseline		**n** **=** **118**	**n** **=** **215**			
			Nephrotoxicity	26 (22.0)	24 (11.2)	0.013	2.25 (1.22–4.13)	NA
Bosso et al. [71]	Aminoglycoside, cyclosporine A, tacrolimus, nonsteroidal inflammatory agents, COX-2 inhibitors, ACEIs, ARB	Nephrotoxicity: increase in S_Cr_ of 0.5 mg/dL or increase ≥50% in S_Cr_ from baseline for two consecutive measurements		**n** **=** **142**	**n** **=** **146**			**n** **=** **280**
			Nephrotoxicity	41 (28.9)	14 (9.6)	<0.001	3.83 (1.98–7.40)	3.64 (1.75–7.59)^b^
Chan et al. [72]	Not stated	Mortality: all cause mortality at hospital discharge		**n** **=** **33**	**n = 39**			
			Mortality	5 (15.2)	6 (15.4)	0.763	0.98 (0.27–3.57)	NA
		Clinical cure: improvement in and resolution of signs and symptoms of VAP, and eradication of MRSA from subsequent BAL or sputum culture after completing ≥7 days of VAN therapy together with CPIS <6 at day 7 of therapy	Clinical cure	24 (72.7)	27 (69.2)	0.948	1.19 (0.43–3.30)	NA
Choi et al. [13]	NA	Nephrotoxicity: increase in S_Cr_ of 0.5 mg/dL or increase ≥50 % in S_Cr_ from baseline		**n** **=** **19**	**n** **=** **37**			
			Nephrotoxicity	2 (10.5)	3 (8.1)	1.000	1.33 (0.20–8.75)	NA
Chung et al. [36]	Not stated	Mortality: overall ICU mortality		**n** **=** **16**	**n** **=** **38**			
			ICU mortality	7 (43.8)	17 (44.7)	0.816	0.96 (0.30–3.12)	NA
		Clinical success: resolution of baseline signs and symptoms of MRSA pneumonia in conjunction with improvement in or lack of progression of chest radiographic abnormalities	Clinical success	8 (50.0)	15 (39.5)	0.680	1.53 (0.47–4.97)	NA
Clemend et al. [37]	Not stated	Mortality: 30-day all-cause mortality		**n** **=** **68**	**n** **=** **26**			
			Mortality	7 (10.3)	4 (15.4)	0.490	0.63 (0.17–2.37)	0.18 (0.03–1.12)^c^
		Clinical success: (1) survived for at least 30 days; (2) eradication of MRSAB within 10 days from the initiation of VAN therapy; or, (3) absence of MRSAB for at least 30 days after discontinuation of therapy	Clinical success	50 (73.5)	21 (80.8)	0.467	0.66 (0.22–2.02)	1.10 (0.26–4.76)^c^
Honda et al. [73]	Not stated	Mortality: any death within 28 days of initial hospital stay; or, if there were no readmission data >28 days after diagnosis of MRSAB available, SSDI was used to verify mortality		**n** **=** **64**	**n** **=** **87**			
			Mortality	13 (20.3)	14 (16.1)	0.650	1.33 (0.58–3.06)	NA
Kullar et al. [33]	Aminoglycosides	Nephrotoxicity: increase in S_Cr_ of 0.5 mg/dL or ≥50 % increase in S_Cr_ from baseline for 2 consecutive measurements		**n** **=** **77**	**n** **=** **141**			
			Nephrotoxicity	10 (13.0)	23 (16.3)	0.648	0.77 (0.34–1.71)	NA
		Clinical success: surviving for at least 30 days, resolution of signs and symptoms of infection at the end of therapy, or absence of MRSAB for at least 7 days of VAN therapy		**n** **=** **86**	**n** **=** **160**			
			Clinical success	52 (60.5)	62 (38.8)	0.002	2.42 (1.41–4.13)	NA
Hermsen et al. [35]	NSAIDs, aminoglycosides (n **=** 12), amphotericin B (n **=** 55), cisplatin, pentamidine, ribavirin, ACE inhibitors, angiotensin receptor blockers, and diuretics	Nephrotoxicity: increase in S_Cr_ of 0.5 mg/dL or increase ≥50 % in S_Cr_ from baseline for at least 2 consecutive measurements		**n** **=** **16**	**n** **=** **39**			
			Nephrotoxicity					
			Definition 1 or 2	5 (31.3)	4 (10.3)	0.103	3.98 (0.91–17.46)	3.27 (0.70–15.25)^d^
			Definition 3	4 (25.0)	5(12.8)	0.422	2.27 (0.52–9.86)	NA
		Mortality: in-hospital mortality	Mortality	3 (18.8)	2 (5.1)	0.141	4.27 (0.64–28.47)	NA
		Clinical success: resolution of signs and symptoms of infection and no additional use of antibiotics or eradication of MRSA, defined as negative culture result at the end of VAN therapy	Clinical success	4 (25.0)	21 (53.8)	0.098	0.29 (0.08–1.04)	NA
Jeffres et al. [31]	IV contrast dye (n **=** 24), loop diuretic (n **=** 49), amphotericin B (n **=** 5), aminoglycoside (n **=** 8), ACE-I or ARB (n **=** 19), cyclosporine or tacrolimus (n **=** 9), vasopressor (n **=** 14)	Nephrotoxicity: increase in S_Cr_ of 0.5 mg/dL or increase in S_Cr_ ≥50 % from baseline for two consecutive measurements		**n** **=** **49**	**n** **=** **45**			
			Nephrotoxicity	27 (55.1)	13 (28.9)	0.018	3.02 (1.28–7.11)	2.82 (1.02–7.74)^e^
Hidayat et al. [30]	Amphotericin B, tobramycin, and tacrolimus (n **=** 27)	Nephrotoxicity: Increase in S_Cr_ of 0.5 mg/dL or increase ≥50 % in S_Cr_ from baseline for 2 consecutive measurements		**n** **=** **63**	**n** **=** **32**			
			Nephrotoxicity	11 (17.5)	0 (0)	0.014	NA	NA

*OR* odds ratio, *95% CI* 95% confidence interval, *VAN* vancomycin, *BSI* bloodstream infections, *S*_*Cr*_ serum creatinine, *CrCl* creatinine clearance, *MRSAB* MRSA bacteremia, *ACEIs* angiotensin converting enzyme inhibitors, *ARB* angiotensin receptor blocking agents, *VAP* ventilator-associated pneumonia, *BAL* bronchoalveolar lavage, *CPIS* clinical pulmonary infection score, *NA* not available, *ICU* intensive care unit, *SSDI* social security death index, *NSAIDs* nonsteroidal anti-inflammatory drugs

^a^Fisher’s exact test or Yates’ continuity correction test

^b^Analysis adjusted for covariates, including age, race, gender, hypotension, receipt of other nephrotoxic agents, length of vancomycin therapy, vancomycin dose per kg of body weight, ICU stay, and comorbidities

^c^Analysis adjusted for covariates, including age, source of bacteremia, duration of bacteremia ≥72 h, ICU care, received effective antibiotic within 24 h of positive blood culture, renal insufficiency (S_Cr_ ≥ 1.3 mg/dL), and vancomycin MIC

^d^Analysis adjusted for APACHE II score

^e^Analysis adjusted for covariates, including IV contrast dye, BUN:SCr ratio > 20, loop diuretic, aminoglycoside, duration of vancomycin ≥14 day, APACHE II score (1-point increments), and vasopressor administration

**Fig. 2:**
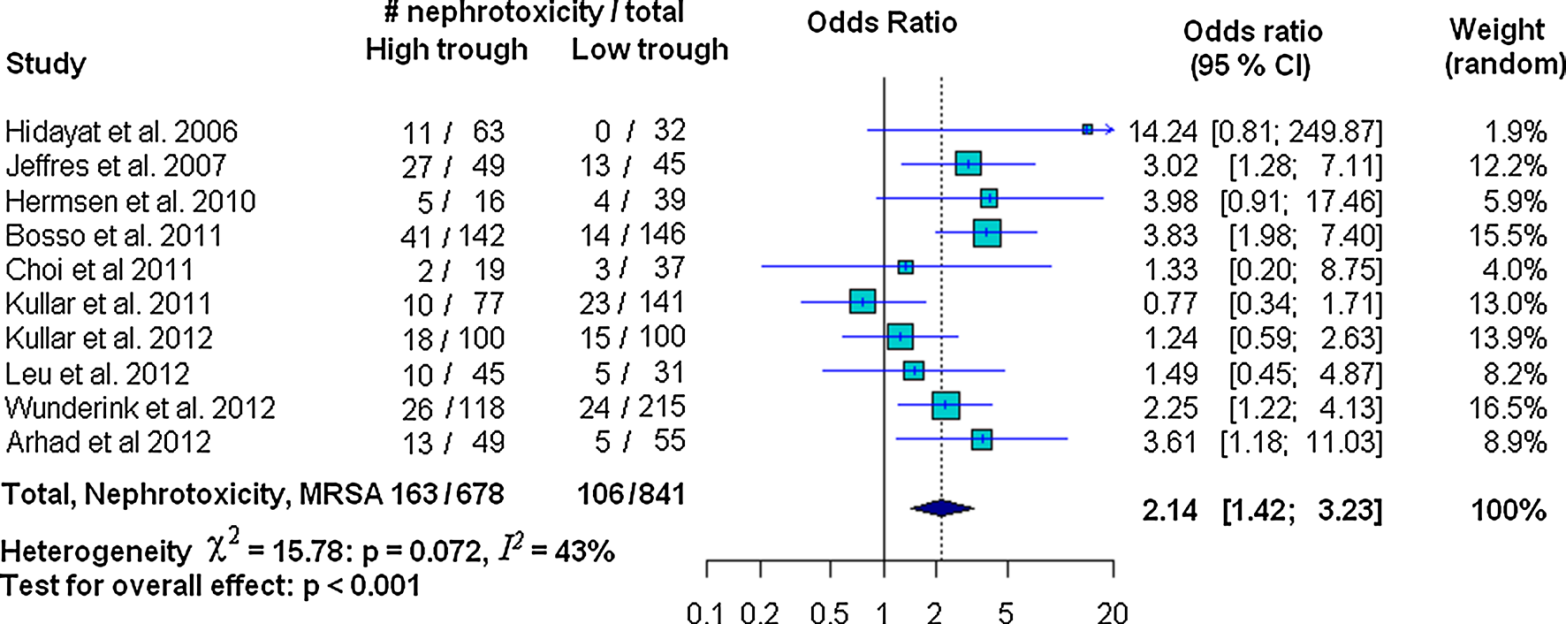
Forest plot of the odds ratio (OR [95 % confidence interval]) for the effect of vancomycin trough levels on nephrotoxicity between *high* and *low* trough levels

**Fig. 3:**
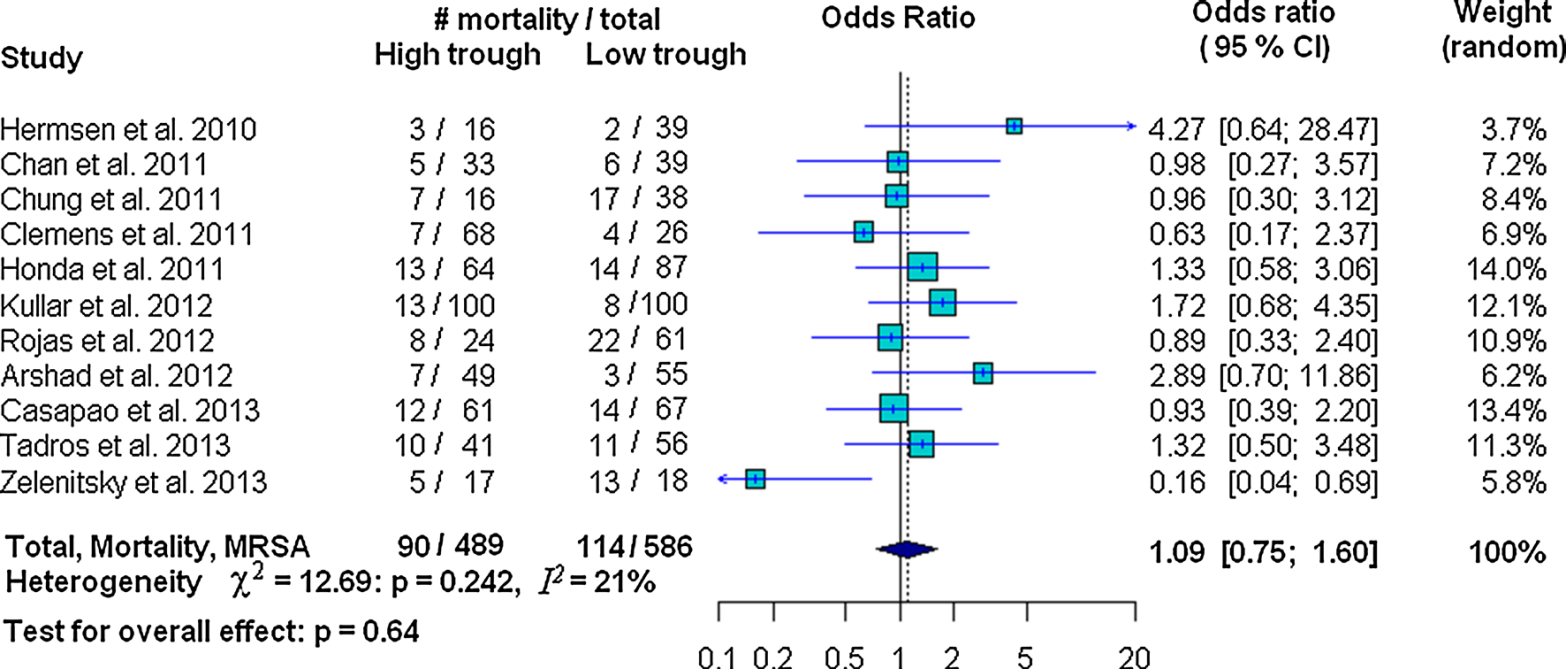
Forest plot of the odds ratio (OR [95 % confidence interval]) for the effect of vancomycin trough levels on mortality between *high* and *low* trough levels

**Fig. 4:**
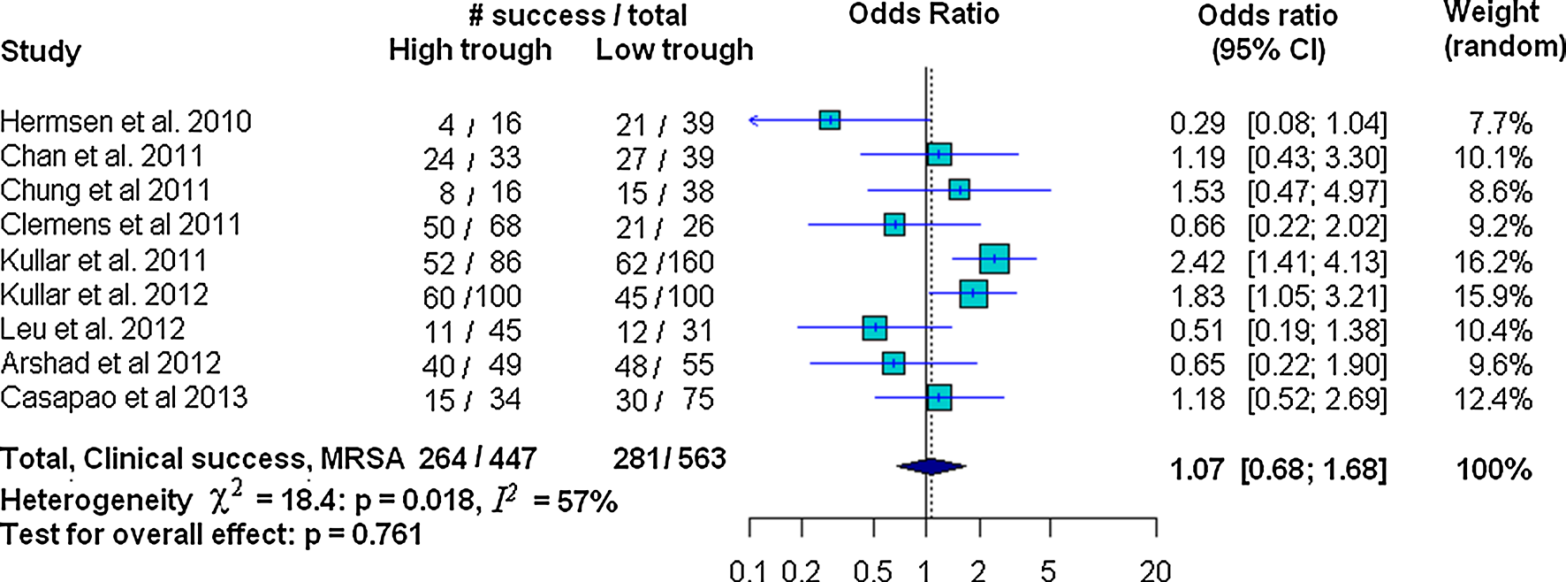
Forest plot of the odds ratio (OR [95 % confidence interval]) for the effect of vancomycin trough levels on clinical success between *high* and *low* trough levels

The amount of heterogeneity among the included studies showed evidence of heterogeneity of ORs across the studies for nephrotoxicity (χ^2^ = 15.78, *p* = 0.072, *I*^*2*^ = 43 % and *τ*^*2*^ = 0.17) and clinical success (χ^2^ = 18.4, *p* = 0.018, *I*^*2*^ = 56.5, and *τ*^*2*^ = 0.248); whereas heterogeneity was not found for mortality (χ^2^ = 12.69, *p* = 0.242; *I*^*2*^ = 21.2, and *τ*^*2*^ = 0.09) (Figs. 2, 3, 4). There was no evidence of heterogeneity of adjusted OR among the three studies used to investigate the association between vancomycin trough levels and nephrotoxicity (χ^2^ = 0.16, *p* = 0.92; *I*^*2*^ = 0 % and *τ*^*2*^ = 0).

In our study, risk of nephrotoxicity was significantly associated with high vancomycin trough levels (OR 2.14 95 % CI 1.42–3.23; *p* < 0.001). There was, however, no evidence of mortality decline (OR 1.09, 95 % CI 0.75–1.60; *p* = 0.64) or improved clinical success (OR 1.07, 95 % CI 0.68–1.68; *p* = 0.761) (Figs. 2, 3, 4). Strength of association between vancomycin trough levels and nephrotoxicity was measured by combining adjusted ORs and confounding variables were adjusted for in each included study (as described in the footnotes of Table 2). After combining the adjusted ORs, the main result was still significant. Specifically, the odds of nephrotoxicity occurring in MRSA-infected patients with trough levels ≥15 mg/L were 3.33 times higher than patients with trough levels <15 mg/L (95 % CI 1.91–5.79; *p* < 0.0001).

**Fig. 5:**
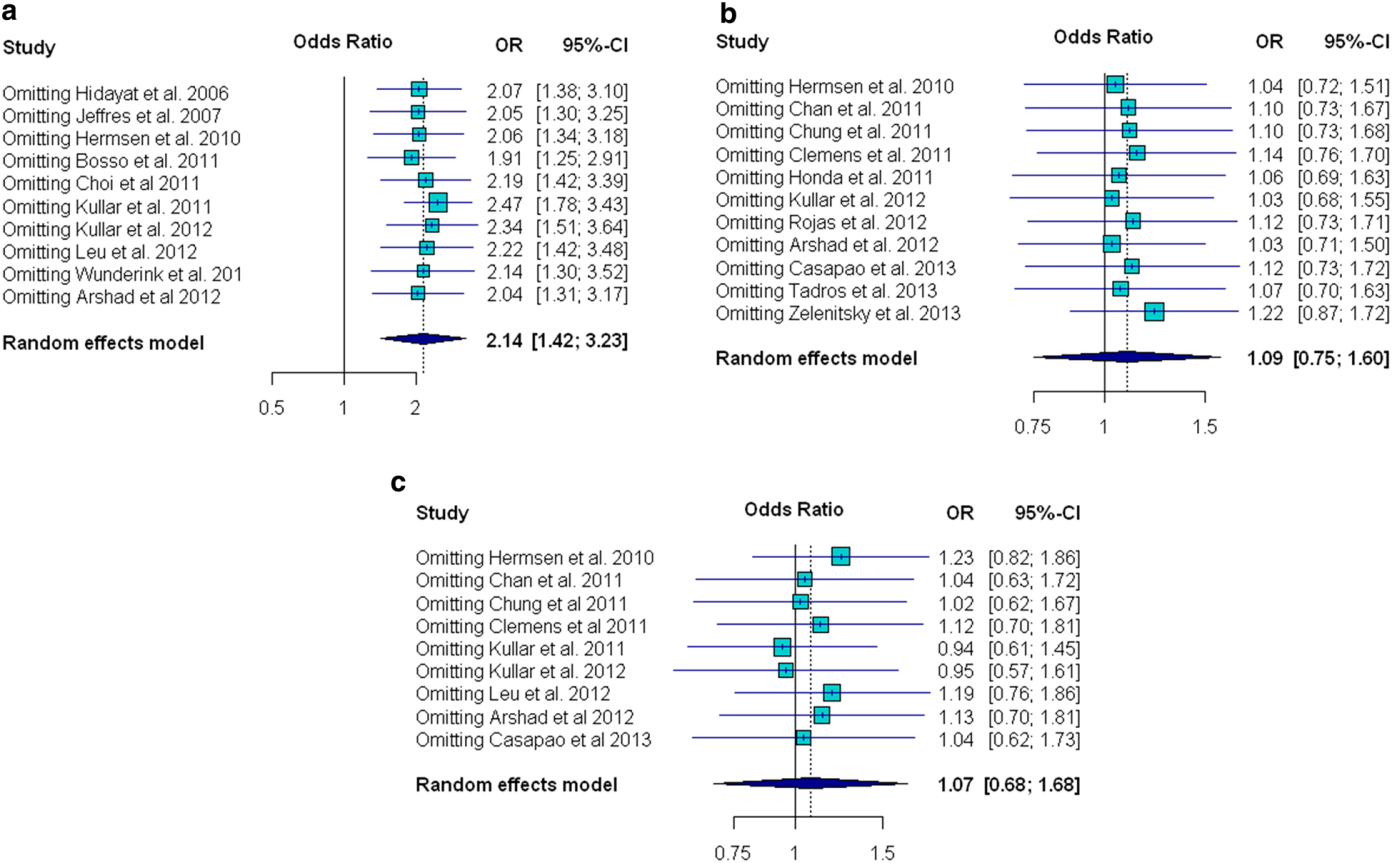
Influence analysis with forest plots of odds ratio (OR) for **a** nephrotoxicity; **b** mortality; and **c** clinical success

For nephrotoxicity, none of the included studies influenced the results to an extent that the conclusion would have changed. The jackknife sensitivity analysis with omitted one study at a time and reevaluated association between trough levels and nephrotoxicity, consistently showed that vancomycin trough levels were associated with risk of nephrotoxicity (Fig. 5a). The sensitivity analysis also showed that the one-by-one exclusion of each study did not affect the conclusion of pooled effect size for either mortality or clinical success.

**Fig. 6:**
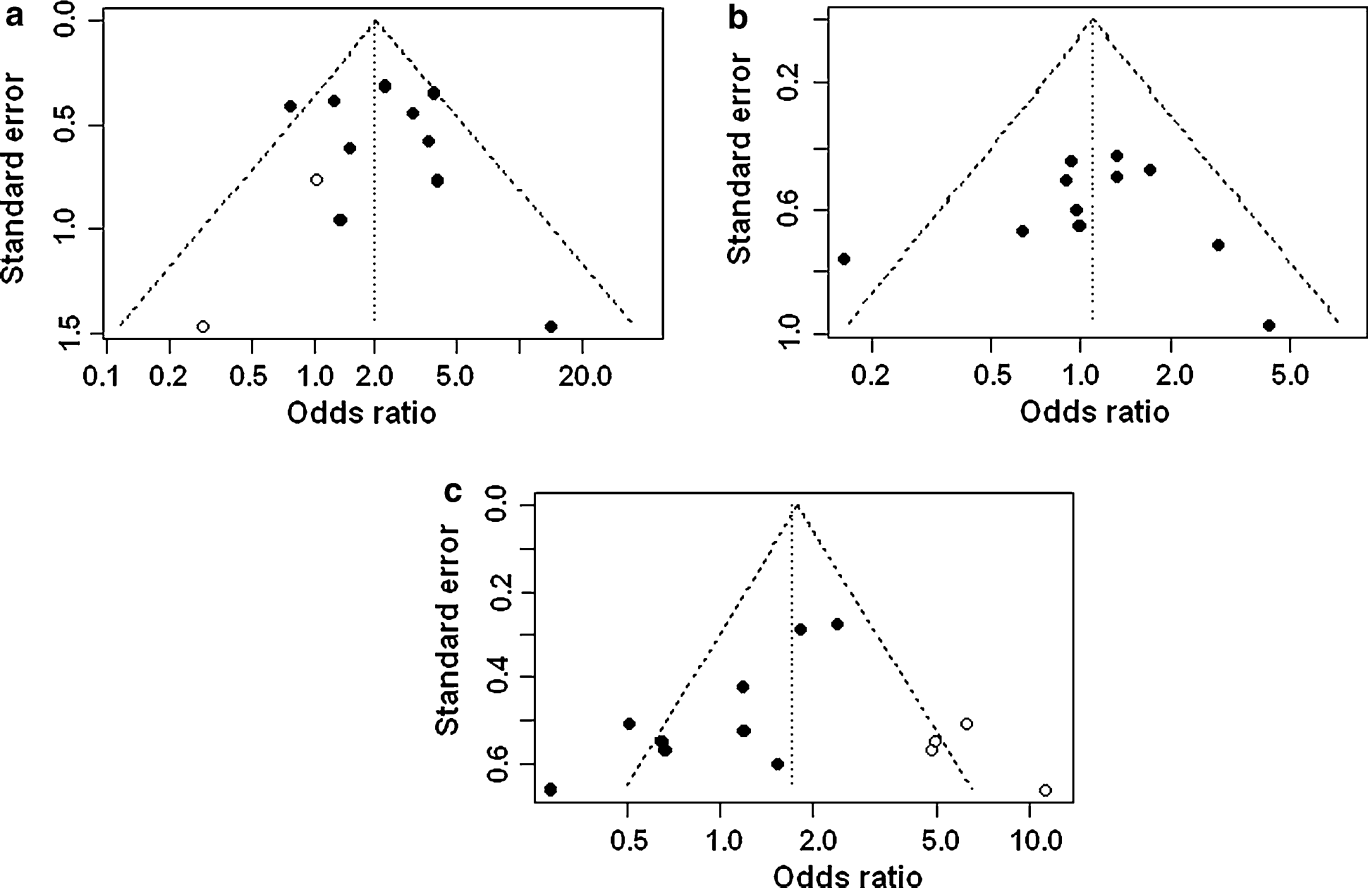
**a** Funnel plot for nephrotoxicity after adjusting for missing studies using the trim and fill method; **b** Symmetrical funnel plot for mortality with an absence of evidence for asymmetry; **c** Funnel plot for clinical success after adjusting for missing studies using the trim and fill method (*filled circles* are original data and *open circles* represent estimated missing studies)

The funnel plot revealed some asymmetry for nephrotoxicity and clinical success (Fig. 6a, c). Formal testing for publication bias relative to two outcomes (nephrotoxicity—Begg’s test: *p* = 0.65 and Egger’s test: *p* = 0.62; and, mortality: Begg’s test: *p* = 0.82 and Egger’s test: *p* = 0.87) did not show statistical significance of asymmetry. These tests, however, yielded statistical significance of asymmetry for clinical success (Begg’s test: *p* = 0.095 and Egger’s test: *p* = 0.003).

Adjustment for funnel plot asymmetry using the trim and fill method did not change the pooled OR for mortality (Fig. 6b). There was some funnel plot asymmetry for both nephrotoxicity and clinical success, and the trim and fill method indicated that the true estimates of pooled OR for nephrotoxicity and clinical success may be 1.99 (95 % CI 1.33–2.96; *p* < 0.001) and 1.71 (95 % CI 1.04–2.81; *p* = 0.034), respectively (Fig. 6a, c). However, after adjusting for funnel plot asymmetry, the conclusion of this meta-analysis regarding nephrotoxicity was not changed, while there was a significant change in the conclusion for clinical success. The trim and fill method demonstrated significant association between high trough levels and clinical success, with the odds of clinical success in MRSA-infected patients with high trough levels being 1.71 times higher than in patients with low trough levels.

## Discussion

Association between vancomycin trough levels and nephrotoxicity was largely uniform across the studies included in this meta-analysis; however incidence of vancomycin-induced nephrotoxicity varied from study to study. Nephrotoxicity rates in the high and low trough groups varied from 18 to 55 % and 0 to 29 %, respectively. This variability was due to varying baseline levels among the study population, length of vancomycin therapy, receipt of other nephrotoxic agents, and renal function (creatinine clearance). From the 19 studies in this analysis, the study in patients with any type of nosocomial MRSA infection had the lowest nephrotoxicity rate [30] while the study in patients with MRSA HCAP had the highest nephrotoxicity rate in both trough level groups [31]. Patients in the study with the highest rate of nephrotoxicity had higher APACHE II scores and received more concomitant nephrotoxic agents than patients in all other studies. These results support the finding of some other studies that concomitant nephrotoxic agents were a risk factor for renal function impairment during vancomycin therapy [30–32]. The meta-analysis by van Hal and colleagues [22] found nephrotoxicity rates that varied from 7 to 67 % in the high trough group (≥15 mg/L) and from 0 to 33 % in the low trough group (<15 mg/L), which both ranges exceeding those from our study. Nephrotoxicity ranges in the van Hal, et al. meta-analysis were wider than the ranges in this study due to some differences of the included studies and no limitation regarding the type of MRSA infection included in their study.

Clinical success was extremely variable across the studies in this analysis with most differences between high and low trough levels being statistically non-significant. There was two exceptions [17, 33], however, with success rates ranging from 24 to 82 % and 39 to 87 % for high and low trough groups, respectively. This variability was due to baseline clinical status of study population and sites of MRSA infections. In the present meta-analysis, the studies with the most patients infected with MRSA pneumonia had low clinical success rates ranging from 24 to 54 % [34, 35]. MRSA-infected patients in the intensive care unit (ICU) were also more likely to have poor clinical success with vancomycin therapy, as compared to non-ICU MRSA-infected patients [36, 37].

Mortality rate also varied widely across the studies in this analysis, ranging from 5 to 20 % in most of the studies and from 36 to 47 % in two studies [36, 38]. No statistically significant differences were observed between high and low trough groups. Patients who had either MRSA bacteremia or who required intensive care had higher mortality rates. The results of this meta-analysis support the results of previous studies [15, 39], all finding that there is no evidence supporting association between higher vancomycin trough levels and improved outcome in patients with MRSA. Although we had originally planned to conduct analysis using a meta-regression model to investigate possible sources of heterogeneity of clinical success among the studies, sufficient data were not available to conduct this analysis.

Based on our review of the literature, this study is the first meta-analysis to compare the safety and efficacy of high vs. low vancomycin trough levels in MRSA-infected patients that were treated according to the current guidelines for treating MRSA infections. The results of this analysis also confirm the results of previous studies that were not included in this meta-analysis, which showed that higher vancomycin trough levels (or higher vancomycin doses) were associated with increase risk of nephrotoxicity [8, 30, 40–46]. Considerable controversy exists concerning the relationship between vancomycin MICs and clinical outcomes. Several studies have reported association between higher vancomycin MIC and increased risk of treatment failure or mortality [30, 47–54], with others finding no significant association with poor outcomes [37, 55–59]. However, recent meta-analysis study in high vancomycin MIC and clinical outcomes in adults with MRSA infections by Jacob and DiazGranados [60] found that high vancomycin MIC was associated with increased mortality and treatment failure.

In this study, the random effects model was used to combine the odds ratios for the outcomes of interest, even though there was no evidence of heterogeneity. The decision to use the random effects model was made a priori, depending upon the nature of the eligible studies and our goals for the following reasons: individual eligible studies were collected from several independent studies; included studies were heterogeneous for study design; and, inferences based on the random effects model can be generalized beyond the studies included in the meta-analysis.

This study has several notable strengths. First, studies that were included in this meta-analysis were independent studies that were conducted during different periods of observation. The results, both individually and collectively, strongly support the fact that there is evidence that higher trough levels are more harmful than low trough levels in terms of nephrotoxicity. Secondly, the results of influence analysis on all outcomes in which each study was removed from the analysis one by one to determine the magnitude of influence on overall effect size, showed that overall effect size was relatively independent of any one particular study. Third, adjusting for asymmetric funnel plots using the trim and fill method did not significantly change the results of this meta-analysis for nephrotoxicity and mortality, indicating that the missing studies were unlikely to have changed the conclusions relating to these outcomes. However, the results of trim and fill analysis showed substantial impact of publication bias on the conclusion for clinical success. Specifically, after trim and fill, the association between high trough level and clinical success was no longer non-significant. Finally, the conclusions of the present meta-analysis were similar to the conclusions from our exploratory meta-analysis that included the excluded 112 patients that had vancomycin trough levels of >20 mg/L.

This study also has some mentionable limitations. First, the definition of mortality and/or clinical success varied slightly among some of the studies included in this meta-analysis, according to the stated protocol of each study. Second, this meta-analysis included a combination of different study designs, including: nine retrospective cohort studies, four prospective cohort studies, two retrospective studies, one non-randomized comparative study, one retrospective quasi-experimental study, one prospective surveillance study, and one multicenter prospective study. Third, only one study met the criteria for combining adjusted OR from different studies for the outcomes of clinical success and mortality; thus, these analyses were not performed. Finally, we did not evaluate the other factors that might associate with high vancomycin trough levels due to lack of information.

The inclusion of grey literature that meets the predefined inclusion criteria of a meta-analysis may help to reduce the effect of publication bias and/or funnel plot asymmetry, whereas the exclusion of grey literature could lead to bias, most notably the overestimation of effect sizes [61, 62]. The research cited in published reviews demonstrates that studies published in journals have a tendency to report larger effect sizes than studies published in grey literature [63, 64].

## Conclusion

Based on pooled adjusted OR, high vancomycin trough level is the variable that was identified as the independent factor associated with risk of nephrotoxicity in MRSA infections. MRSA-infected patients with trough levels ≥15 mg/L had greater odds of nephrotoxicity than those with trough levels <15 mg/L. There was no evidence of difference in mortality or clinical success between patients with trough levels ≥15 and <15 mg/L. However, we need to acknowledge that this conclusion does not take into account vancomycin MIC data, which were not available for analysis in this study. Since adjustment of funnel plot asymmetry using the trim and fill method yielded significant change in pooled OR for clinical success, association between high vancomycin trough levels and both risk for adverse events and improvement in clinical outcomes in patients with MRSA infection requires further study.

## Additional file

10.1186/s13104-016-2252-7 Supplementary data.

## Abbreviations

MRSA: methicillin-resistant *Staphylococcus aureus*
MIC: minimum inhibitory concentration
