# Supplementary material for: The safety and efficacy of high versus low vancomycin trough levels in the treatment of patients with infections caused by methicillin-resistant Staphylococcus aureus: a meta-analysis
Source: BMC Res Notes. 2016 Sep 29;9:455. doi: 10.1186/s13104-016-2252-7 (PMC5041442; doi:10.1186/s13104-016-2252-7)
Supplement: Supplementary file 1 — 10.1186/s13104-016-2252-7 Supplementary data. [file 13104_2016_2252_MOESM1_ESM.doc]

**Research Plan**

**Principle investigator**: ST

**Hypotheses:** High vancomycin trough levels induce nephrotoxicity in patients with MRSA infection.

Higher vancomycin trough levels are associated with clinical outcomes (i.e., clinical success, mortality) in MRSA-infected patients.

**Primary endpoint:** Nephrotoxicity.

**Secondary endpoint**: Mortality, clinical success.

**Search strategy**

Databases: MEDLINE/ Pubmed, Web of Science, and Scopus

Conference proceedings: Infectious Diseases Society of America (IDSA), International Society for Infectious Diseases (ISID), American Society for Microbiology (ASM), and European Society of Clinical Microbiology and Infectious Diseases (ESCMID).

[www.idsociety.org](http://www.idsociety.org/), [www.isid.org](http://www.isid.org/), [www.asm.org](http://www.asm.org/), and www.escmid.org Accessed 31 Oct 2013.

**Search terms**

The search terms used for PubMed were as follows: 1) “vancomycin trough level” AND “methicillin-resistant *Staphylococcus aureus*”, 2) “vancomycin trough levels” AND “methicillin-resistant *Staphylococcus aureus*”, 3) “vancomycin trough concentration” AND “methicillin-resistant *Staphylococcus aureus*”, and 4) vancomycin AND nephrotoxicity AND “methicillin-resistant *Staphylococcus aureus*”. The search terms used for ISI Web of Science were the following: 1) vancomycin AND “trough level* or "trough concentration" and "methicillin-resistant *Staphylococcus aureus*" and 2) vancomycin and nephrotoxicity and “methicillin-resistant *Staphylococcus aureus*”. The search terms used for Scopus were as follows: 1) vancomycin AND "methicillin-resistant *Staphylococcus aureus*" AND "trough level", 2) vancomycin AND "trough concentration" AND "methicillin-resistant *Staphylococcus aureus*", and 3) vancomycin AND "trough level" AND nephrotoxicity AND "methicillin-resistant *Staphylococcus aureus*". MeSH terms for “vancomycin” and “methicillin-resistant *Staphylococcus aureus*” were also included in the PubMed search. Accessed 31 Oct 2013.

**Process:**

1. ST and PK discussed individual studies to decide about inclusion or exclusion. ST collected all abstracts and review abstracts.
2. ST reviewed full-text articles and conference abstracts of selected studies for further evaluation whether appropriate to include the studies in the meta-analysis.
3. ST and PK met to discuss the data extraction findings and characteristics of each included study

**Data extraction**: the first author's last name, year of publication, study location, patient’ characteristic, study design, sample size (number of subjects in high and low trough groups), vancomycin trough measurement, duration of vancomycin therapy, other nephrotoxic drug used, outcome definition, ORs with 95% CIs for each outcome, and covariates adjusted for in the multivariable model.

**Data synthesis**

Random effects models were used in order to expect considerable heterogeneity among the included studies, which were mostly observational studies.
